# Chemical shift assignment of the alternative scaffold protein IscA

**DOI:** 10.1007/s12104-016-9672-0

**Published:** 2016-02-18

**Authors:** Matija Popovic, Annalisa Pastore

**Affiliations:** Maurice Wohl Institute, King’s College London, 5 Cutcombe Rd., London, SE5 9RT UK

**Keywords:** Iron metabolism, Iron sulphur clusters, Metalloproteins, Scaffold protein, Structure

## Abstract

The IscA protein (11.5 kDa) is an essential component of the iron sulphur cluster biogenesis machine. In bacteria, the machine components are clustered in operons, amongst which the most important is the isc operon. Bacterial IscA has direct homologues also in eukaryotes. Like the protein IscU, IscA is thought to assist cluster formation as an alternative scaffold protein which receives the cluster before transferring it further to the final acceptors. Several crystal structures have been published. They all report an IscA dimeric form, although the packing of the protomers in the dimers differs amongst structures. No solution studies have currently been reported. Here we report the ^1^H, ^13^C and ^15^N backbone and side-chain chemical shift assignments of the cluster-free *E. coli* IscA as a starting point for further studies of the structure and functions of this still poorly characterized protein. We show that IscA exists in solution as an equilibrium between different species. Spectrum assignment was thus challenging given the heterogeneous nature of the sample but doable through judicious choice of selective labelling and concentration dependent studies.

## Biological context

In biology, iron-sulfur clusters are prosthetic groups attached to proteins which provide the cell with an important source of redox potential (Beinert et al. 1997). Iron-sulphur cluster proteins play essential roles in several different pathways, including the Krebs cycle, purine metabolism, gene regulation and several others (Johnson et al. 2005; Xu and Moller 2011; Fontecave 2006). Their assembly is tightly regulated since iron is readily oxidized and can generate free radicals. Sulphur is also a highly toxic elements in several of its forms. To solve this problem and allow an efficient assembly, specific machines have evolved to assist iron-sulfur cluster assembly (for a recent review see Mettert and Kiley 2015). In bacteria, these machines are grouped in operons (Zheng et al. 1998). The *isc* operon is the central genetic locus for the pathway as it contains genes highly conserved also in high eukaryotes (Lill 2009). A group of about ten proteins are encoded in the operon, amongst which is the promoter IscR, the desulfurase IscS, the scaffold IscU, the two putative chaperones HscA and HscB, a ferredoxin and IscA (Roche et al. 2013). While the role of some components is relatively clear, the exact function of IscA remains uncertain. IscA is a relatively small protein which contains three conserved cysteines. It was shown to bind both iron and be able to host the cluster (Zeng et al. 2007; Ding et al. 2007). Cluster loaded IscU readily donates it to IscA suggesting that the latter protein has a higher affinity to the cluster. It was thus proposed to be an alternative scaffold protein (Krebs et al. 2001; Ollagnier-de-Choudens et al. 2001; Bonomi et al. 2005). However, it remains unclear why a second scaffold should be necessary and at which step is IscA required. Different crystal structures of IscA have been solved (Bilder et al. 2004; Cupp-Vickery et al. 2004; Morimoto et al. 2006). They all have shown that IscA is a dimer but the interface between protomers is different in some of the structures leaving open the question of which species exists in solution. In one of the structures the resulting packing is non-symmetric. No solution structure exists yet which could clarify this question and provide more information about the function. Here, we report the backbone assignment in solution of IscA as a first step to the characterization in solution of the IscA aggregation state and its interactions with other proteins.

## Materials and methods

### Protein production and purification

The recombinant *E. coli* IscA (107 residues) was over-expressed in the *E. coli* host strain BL21 (DE3) using a kanamycin resistant pETM30 vector. Isotopically ^15^N- and ^13^C/^15^N-labelled samples were expressed in minimal (M9) medium supplemented with ^15^N-ammonium sulphate and ^13^C-glucose as the sole sources of nitrogen and carbon respectively. Purification was performed with a three-step protocol which used anion exchange and size exclusion chromatography. In short, the frozen cell pellet was re-suspended in the lysis buffer (20 mM Tris–HCl, 0.2 % IGEPAL, 20 mM DTT, pH 7.5 containing EDTA-free protease inhibitor cocktail tablet (Roche) and sonicated. After centrifugation, the supernatant was loaded on a weak anion exchange column (HiTrap DEAE, GE Healthcare) and eluted with a 0–250 mM NaCl gradient. The crude material containing the protein was diluted to reduce the salt concentration and loaded on a strong anion exchange column (HiTrapQ, GE Healthcare) and eluted with 0–500 mM NaCl gradient. The eluted protein (~70 % pure) was gently concentrated using the stirred-cell ultrafiltration system (Amicon, Millipre) and further purified by FPLC size exclusion chromatography (HiLoad 16/600 Superdex 75pg, GE Healthcare). Protein identity and purity was tested by PAGE and mass spectrometry. Because containing only one tyrosine, IscA has a low estimated extension coefficient (1490 M^−1^ cm^−1^). Concentrations were estimated by the Bradford assay using a calibration curve created with BSA samples of known concentrations.

## NMR spectroscopy

NMR spectra for resonance assignments were acquired on samples containing ^15^N- or ^15^N,^13^C-labelled IscA (0.04–0.5 mM) in 20 mM Tris–HCl pH 7.5, 150 mM NaCl, 5 mM TCEP, 8 % ^2^H_2_O. The spectra were typically recorded at 298 K using 600, 700, 800 and 950 MHz frequency spectrometers, all equipped with triple resonance gradient CryoProbes. HNCA, HN(CO)CA, HNCO, HN(CA)CO, HNCACB and HN(CO)CACB experiments (Muhandiram and Kay 1994) were recorded in combination with a 3D ^15^N-edited NOESY-HSQC experiment to carry out spectral assignment. All spectra were processed using NMRPipe/NMRDraw software (Delaglio et al. 1995) and analyzed using CCPN software (Fogh et al. 2002).

## Resonance assignment and deposition

**Fig. 1:**
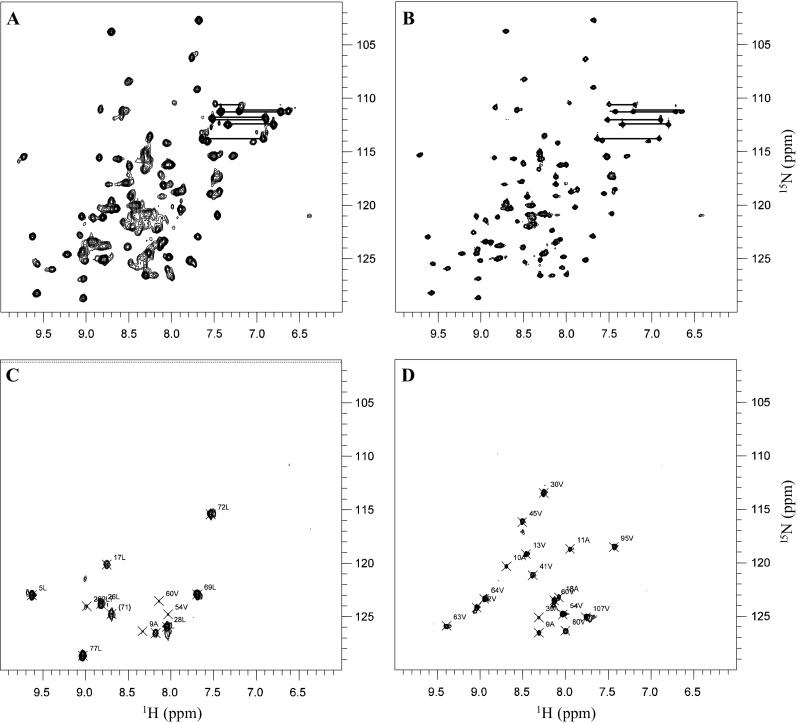
^1^H, ^15^N-HSQC spectra of the *E. coli* IscA protein recorded at 298 K on 600 MHz spectrometer. **a** ^15^N-uniformely labelled spectrum at a protein concentration of 200 μM. **b** ^15^N-uniformely labelled spectrum under diluted conditions (40 μM). **c** Selectively ^15^N-leucine labelled spectrum at 100 μM and **d** ^15^N-valine selectively labelled spectrum at 80 μM. Scrambling to alanine and valine respectively were observed in the valine and leucine ^15^N selectively labelled spectra. Peak doubles are indicated with *primed numbers*. The side chains of glutamines and asparagines are indicated by *connecting lines*

The overall excellent chemical shift dispersion in IscA HSQC spectra is indicative of a well folded protein. However, all protein samples and especially those above 100 μM contained more amide resonances from the expected 118 (Fig. 1a, b). Typically, also the spectra with low heterogeneity contained an excess of amide resonances (~136 peaks including side chain resonances). Most of the extra resonances were less intense than others or their intensities strongly varied with the concentration. After extensive characterization with size exclusion chromatography we concluded that the observed behaviour is caused by formation of dimers and higher species.

In order to reduce protein oligomerizaton we prepared samples in 20 mM Tris–HCl at different salt concentrations (NaCl 0–1000 mM), pH values (6–8) and high reducent agent concentrations (either 20 mM DTT or 5 mM TCEP). These conditions were kept throughout purification. Nevertheless, the size exclusion chromatograms showed no significant differences when performed at 50, 150 or 1000 mM NaCl concentrations. Accordingly, we did not observe reduction of the number of amide resonances in spectra acquired at different NaCl concentrations (50, 150 and 250 mM) or at different pH values (6.0, 7.0, 7.5 and 8).

The best results were obtained when purification was performed by keeping the protein concentration below 150 μM all the way through purification. This behaviour is well in agreement with the available crystal structures of IscA which all indicate dimer formation but also indicates that the different species are in equilibrium with each other within the range of concentrations observed in the NMR samples. The best NMR sample in terms of spectral homogeneity was recorded on a 40 μM sample directly obtained from size excluded chromatography selecting the sharp peak corresponding to the molecular weight of the monomer (Fig. 1b). This ^15^N HSQC spectrum had to be recorded with 128 scans to obtain sufficient signal-to-noise ratio. Unfortunately higher minimal concentration is needed for recording 3D spectra. For this purpose, we had to use ~200 μM samples. At this concentration, 160 of the expected 118 resonances were observed but their intensities were greatly differing reflecting populations. It is however important to stress that direct correlation between resonance intensity and populations in HSQC spectra is not possible since intensity is also strongly regulated by the flexibility of the specific amide group. We are not new to this behaviour as we had previously encountered a similar difficulty in the assignment of the AXH domain of ataxin-1 (de Chiara et al. 2013). To overcome the difficulties and properly trace the connectivities, we used various concentrations and several dilution/concentration experiments which allowed us to follow the heterogeneity of the sample. We also used selectively labelled schemes feeding the cells with specific labelled amino acids (i.e. ^15^N valine and leucine labelled samples; Fig. 1c). These experiments confirmed and rectified previous assignment directing to a fully consistent solution tracing the most persistent and intense species.

Almost full backbone assignment of ^1^H, ^13^C and ^15^N of IscA was obtained as described below. Sequence specific ^1^HN, ^15^N, ^13^Cα, ^13^Cβ and ^13^C assignment of the IscA spectrum was obtained using HNCA, HN(CO)CA, HNCO, HN(CA)CO, HNCACB and HN(CO)CACB experiments (Muhandiram and Kay 1994) in combination with a 3D ^15^N-edited NOESY-HSQC experiment. We managed to assign 87 % of the backbone HN connectivities and 91, 84 and 83 % of the Cα, Cβ and C’ resonances respectively. Residues S2, G34, C35, S36, G37 could not be assigned because not observable in the spectra even though they should have well distinguishable Cα and/or Cβ resonances. We encountered a similar situation with L84 which could not be assigned even in the ^15^N-leucine selectively labelled spectrum. Residues R31, D46, E47, K81, N85, E86, F90 and N92 could only be tentatively assigned because of the strong signal overlap and given that their sequential connectivities are interrupted by proline residues.

The ^1^H, ^13^C and ^15^N chemical shifts have been deposited to the BioMagResBank (BMRB) database and are available under the accession number 25912.

**Fig. 2:**
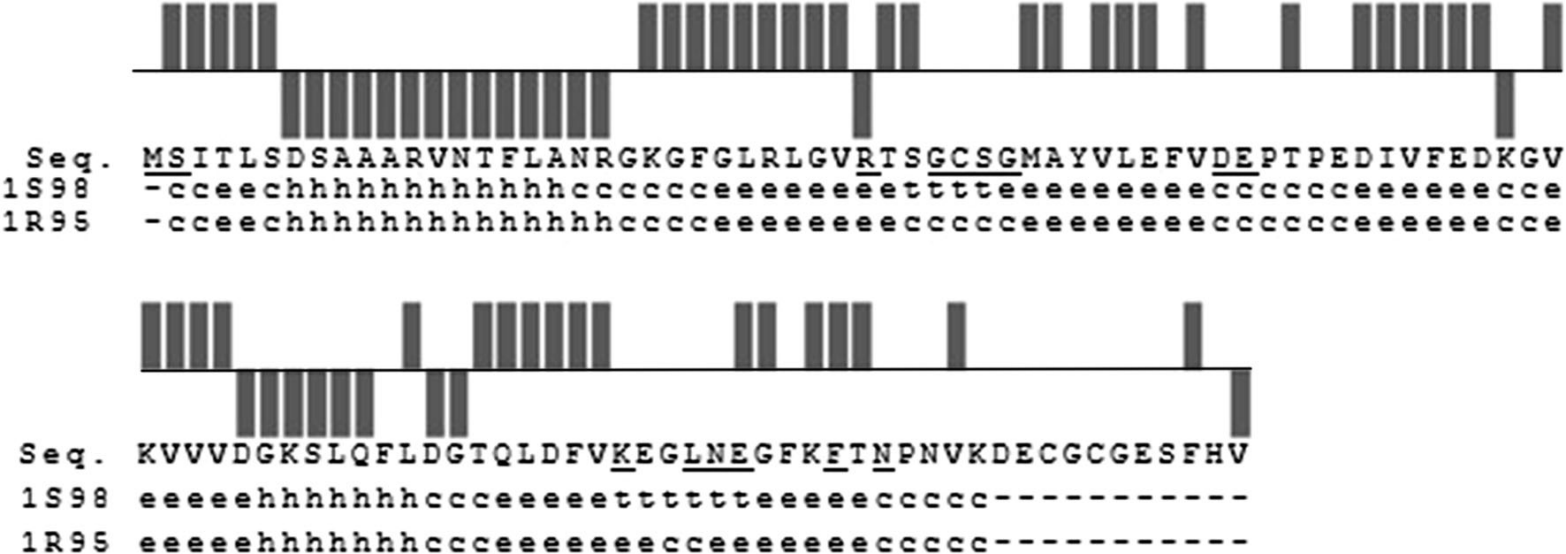
Chemical shift indices obtained from C′ Cα and Cβ chemical shifts. The secondary structure indicated by these values is compared to that of two X-ray structures (1S98 and 1R95). Residues with non-assigned NH crosspeaks are *underlined*; regions not observed in the crystal structures are indicated as *dash lines*. Secondary structure indications use the Kabsh and Sander convention (Kabsch and Sander 1983)

Secondary structure was predicted on the base of the ^13^C chemical shifts using CSI calculation method (Wishart and Sykes 1994) integrated in the CCPN analysis program (Vranken et al. 2005; Fig. 2). Comparison with that of the X-ray structures 1S98 and 1R95 shows an excellent agreement. Overall, these results put us in an excellent position to understand the specific role of IscA in iron-sulfur cluster biogenesis and to study the interactions between IscA and other proteins.
